# Supplementary material for: Senescent cell heterogeneity and responses to senolytic treatment are related to cell cycle status during senescence induction
Source: Aging (Albany NY). 2025 Aug 7;17(8):2063–78. doi: 10.18632/aging.206299 (PMC12422821; doi:10.18632/aging.206299)
Supplement: Supplementary Figures [file aging-17-8-206299-s001.pdf]

SUPPLEMENTARY FIGURES

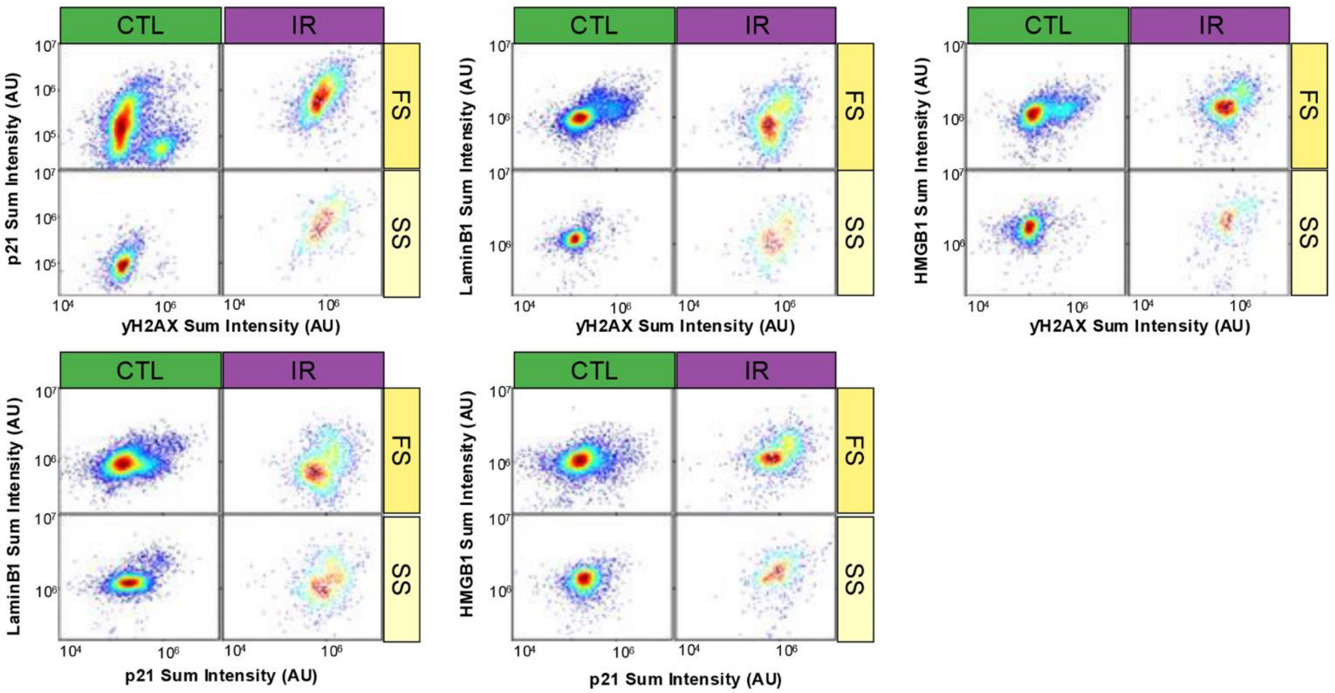

Supplementary Figure 1. Differential expression of senescence markers reveals two distinct populations. Density scatter plots display the expression levels of co-stained senescence markers in individual cells identified through immunofluorescence. The markers include P21 vs. γH2AX, Lamin B1 vs. γH2AX, HMGB1 vs. γH2AX, Lamin B1 vs. P21, and HMGB1 vs. P21 at the single-cell level under both IR and control conditions in either full serum (FS) or serum-starved (SS) culturing conditions. Colors indicate the density regional distribution within the scatter plot.

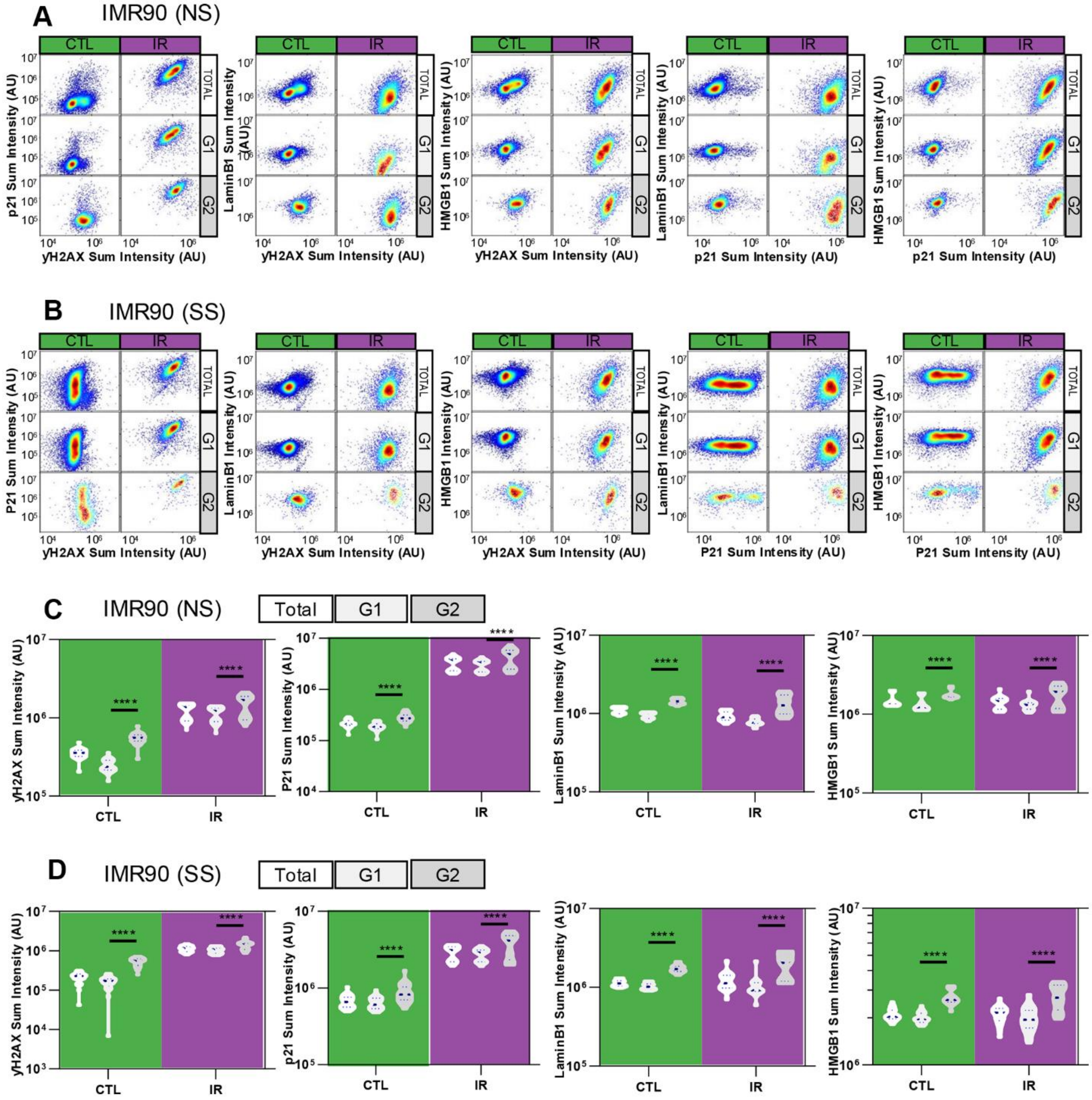

Supplementary Figure 2. Differential expression of senescence markers in G1 and G2 senescent IMR-90 fibroblasts. (**A**, **B**) Scatterplots with senescence markers co-staining data from IMR-90 fibroblasts. Scatterplots show data for the overall cell population (white), G1 subpopulation (light-grey), or G2 subpopulation (dark grey). Data from full serum (FS) culturing conditions is shown in (**A**), while serum-starved (SS) data is shown in (**B**). (**C**, **D**) Violin plots presenting the average expression levels of various senescence markers from replicated wells and experiments within the overall population, G1 or G2 subpopulations. Data from FS culturing conditions is shown in (**C**), while SS data is shown in (**D**). A one-way ANOVA statistical test was performed where ****: p-value < 0.0001.
